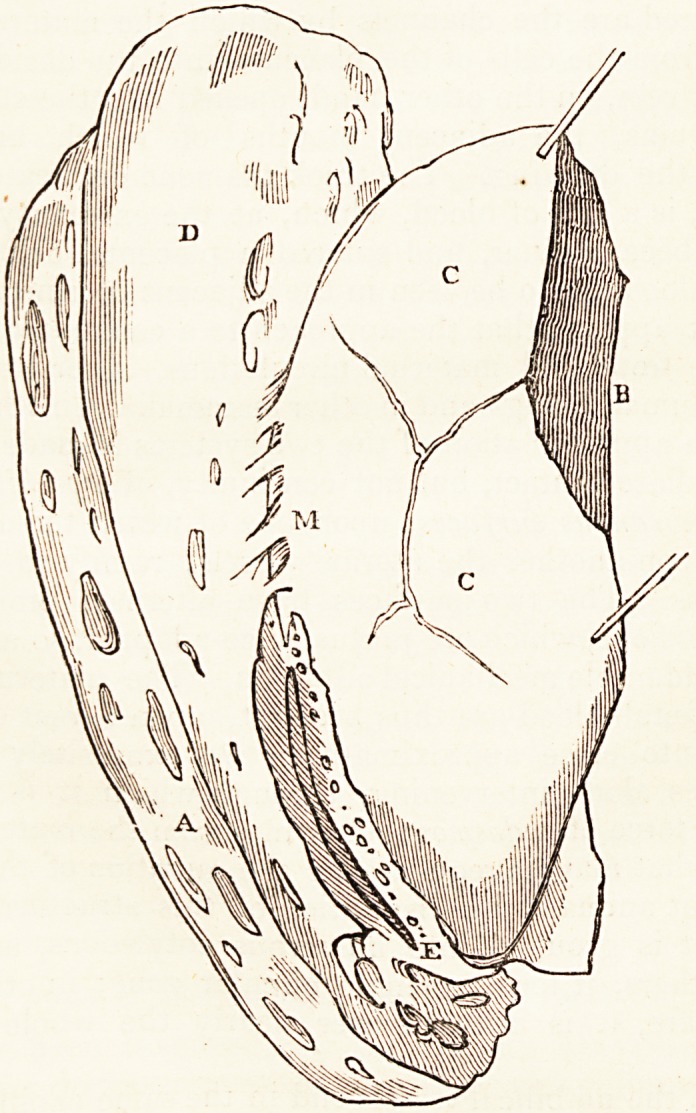# Structure of the Placenta. Examination of the Hunterian Preparations at the College of Surgeons

**Published:** 1835-04-01

**Authors:** 


STRUCTURE OF THE PLACENTA. *Examination of the* HUNTERIAN PREPARATIONS *at the College of Surgeons.* From the *Medical Gazette; with additional Remarks, by* MR. MAYO.

As the structure of the human placenta has lately excited much inquiry, the following statement is offeredfor insertion to the Editor of the "Medical Gazette." It is an account of an examination of the Hunterian preparations relating to this subject, in the museum of the Royal College of Surgeons in London.

The preparation in the Hunterian museum which throws the most light upon the structure of the placenta, and upon the extension of the maternal circulation into it, is marked No. 3535.

The specimen is a triangular portion of a placenta, having a superficies of about four square inches, one of the sides of which is formed by the margin of the placenta, the other two being cut surfaces, the depth of which, at the angle at which they meet, is an inch and a half. It consists of one entire lobe, and of portions of three other lobes of the placenta. Three kinds of wax injection (one yellow, a second red, a third black,) have been thrown into it. The yellow wax, which appears to have been injected last, and more sparingly than the others, is seen to be in the umbilical arteries. The sources and place of the black and of the red injection, with the latter of which the portion of placenta under consideration is most coloured, will be pointed out afterwards.

The substance of the placenta is seen to be covered by two layers of decidua, one disposed on its uterine, the other over its fœtal surface: these two layers of decidua meet, of course, and become *one*, at the circumference of the placenta. Upon one of the cut surfaces of the placenta, productions of the decidua are seen extending through the placenta, from the fœtal to the uterine layer of the decidua, which they unite.

Upon the uterine surface of the uterine layer of the decidua are seen orifices of different sizes, some containing red wax, others black wax. Some of these orifices are upon the surface of the lobes, others at the interlobular spaces. The orifices containing red wax open indiscriminately at either situation. The orifices containing black wax open principally at the interlobular spaces. It may be presumed that the orifices containing black wax were continuous with, and injected from, the uterine veins; and that those which contained red wax were continuous with, and injected from, the uterine arteries, upon the following grounds:

The orifices containing black wax are larger, and lead into larger channels, than those which contain red wax. Some of those which contain red wax lead into channels which have the singular tortuous character, described by Mr. Hunter and others as characterizing the termination of the uterine arteries. And there is a preparation of part of an uterus, in the same series in the gallery, which there can be little doubt is that from which the specimen under consideration was separated, and in which the arteries are injected with red, the veins with black wax.

The orifices upon the uterine surface of the uterine layer of the decidua lead into flattened tubes of greater or less length, which tubes appear to be regular channels, with smooth internal surfaces, formed in the substance of the productions of the decidua. Of these tubes, those which contain red wax are called, in the following description, *decidual arteries;* those which contain black wax, *decidual veins.*

One large decidual vein runs along the placental margin of one lobe. Another, of smaller size, passes nearly vertically in an interlobular fissure from the uterine to the fœtal surface of the placenta. The former terminates opposite to an interlobular space at the edge of the placenta, in two small decidual veins: one of these smaller veins opens into the extremity of the vertical interlobular vein just described; the other extends along the fœtal surface of the placenta. A third decidual vein, smaller than either of the preceding, dips into a different interlobular space, and, after a course of a quarter of an inch, divides into two smaller veins.

Of the decidual arteries, those which open upon the lobules of the placenta make a sudden turn below the uterine layer of the decidua, and terminate there, forming the short curling arteries of Hunter. The interlobular decidual arteries descend nearly vertically towards the fœtal surface of the placenta. One is seen to reach that surface, accompanying an interlobular decidual vein, described above. Another, larger than the preceding, passes, for the length of half an inch only, into an interlobular space.

This preparation, therefore, distinctly establishes that there exist, formed in the decidua, one terminating on, others extending into, or through the substance of, the placenta, regular channels; one set of which is continuous with, and receives blood from, the uterine arteries; while the other is continuous with, and returns blood to the uterine veins.

The manner in which the decidual vessels terminate, is best seen in those decidual arteries and veins which enter the substance of the placenta, but do not extend to its fœtal surface. Each of the vessels of this class, that was examined, divides into two branches. These branches, after a short straight course, terminate abruptly. At their abrupt terminations, the tissue of which they are composed appears, at more than one point, to be porous. The lining of the decidual trunks does not appear entirely divested of the same character, but in parts presents smooth and regular openings. This appearance in the decidual trunks is most distinctly seen in a large interlobular decidual vein. Immediately without and around the tissue in which the vascular channels are formed, is the injected and seemingly cellular decidual tissue of the placenta.

The preparation, No. 3535, would indeed leave it in doubt whether the red injection, with which it is coloured, is contained in cells, or in a series of minute decidual tubes, comparable to capillaries. But there are four other preparations in the Hunterian museum, seemingly taken from the same subject with that described, and in which the portions of uterus and placenta are not separated. Three of these, Nos. 3539, 3533, and 3538, and especially the first, certainly display a series of cells filled with black injection from the uterine veins. In one of these, numerous openings into cells from the side of a marginal decidual vein are distinctly to be seen.

There are other preparations which, taken singly, are less illustrative; but the whole beautiful series appears to us to establish, in the clearest manner, the correctness of the views which Hunter entertained of the relation of the maternal to the fœtal circulation in the human placenta.

EDWARD STANLEY.
HERBERT MAYO.

The preceding account of an examination of one of the Hunterian specimens was drawn up by Mr. Mayo, in the presence of Mr. Stanley and Mr. Owen; and all three concurred in thinking that the preparation established all the points of structure which are described in it. The subject, however, is one upon which physiologists are not yet entirely agreed; and any additional evidence upon it may therefore be acceptable to the reader. The diagram which immediately follows has been made with this view: it was drawn by Mr. Mayo, from a preparation, which he put up for the King's College museum. The preparation is a slice of a gravid uterus at the full time, with the corresponding slice of placenta, which is partially detached. The vessels of the uterus are uninjected. The umbilical arteries and veins are injected with size and vermilion. In the figure, A represents the section of the uterus; D, the placental surface of the uterus; C, the uterine surface of the slice of placenta; B, the section of the placenta.

In separating the placenta from the uterus, the decidual adhesions of the one to the other were found to be very numerous: some of these are represented at M. They appear at first sight membranous bands only, but many of them are certainly deciduous channels, i. e. tubes in the decidua, which are continuous, on the one hand, with the arteries and veins of the uterus, and, on the other, open into the placental cells. They are the channels called, in the preceding paper, *decidual arteries* and *veins*. The letter E in the figure represents one of the largest of these, a decidual vein, running along the margin of the placenta: the numerous orifices in it open into regular cells; the orifices being of different sizes, the surface of the production of deciduous membrane forming the cells being smooth, and resembling that of the air-cells of the lungs in cold-blooded animals. The little that is seen of the surface of the cells has an appearance of vascularity, from the injected capillaries of the umbilical system. The openings figured are the channels by which the maternal blood escaped from the cells of the placenta into the decidual vein, E. That vein, on the other hand, opened into the contiguous uterine veins, the adjacent mouths of which are represented in the drawing. The thick filament delineated in the vessel, E, is a clot of blood, which, at the extremity furthest from E, became finer, and entered a placental cell: similar clots of blood are to be seen in the adjacent uterine veins.

Thus it appears that the approach to a communication between the fœtal and maternal circulations, is very nearly the same in human beings and in other mammalia. In other mammalia, the approximation of the two systems is made through the immediate contact, but not continuity, of *two highly vascular membranous surfaces*; upon one of which the umbilical vessels, upon another the uterine vessels, ramify to capillary minuteness. The two surfaces have alternate prominences and depressions, which are mutually co-adapted, so as to produce considerable mechanical adhesion. The maternal blood and the fœtal blood are thus brought, upon a vast extent of surface, into close approximation; two exquisitely delicate membranes alone intervening, through which it is easy to suppose a force of *endosmose*, drawing from the maternal circulation what may be requisite for the nutrition of the fœtus. In different animals the disposition of this structure varies: in some it is grouped into numerous cotyledons, as in the cow; in others, it forms a single circular zone; in others, as in the mare, it is diffused over nearly the whole uterine surface.

In man, the umbilical vessels end in the same capillarity as in other mammalia, but *that* is not applied against another capillary surface: the umbilical capillaries are distributed over the walls of cells, formed for that purpose in the decidua, and comparable to pulmonary cells, or to the spongy texture of the penis, into which the uterine arteries pour blood, and from which the uterine veins receive it. The membranes interposed are equally fine as in the case of other mammalia; but the approximation is not of capillary to capillary, but of capillary to cell. The apposition, too, is not of separable surfaces: the placenta, with its cells, is an expansion of the decidua; between the two separated layers, united as they are by numerous cross septa, the placental cellular tissue is laid out, and the umbilical vessels from the fœtal body, piercing the fœtal surface of the placenta, ramify among the placental cells with so intricate and involved a distribution among and around the cells, that the extrication of the one from the other is impossible. In animals, the two opposed membranous surfaces constitute a separable maternal and fœtal system; in the human placenta, the fœtal and maternal systems are separable in idea only. There exists, no doubt, a maternal as well as a fœtal portion of the placenta; but they do not occupy different aspects of the placenta, but are equally and entirely commingled throughout.

In thus contrasting the *animal* with the *human* structure, it has been assumed that the organization in the pregnant monkey corresponds with that in the horse or cow: this, however, has not yet been established. What would incline one to expect it, is, that monkeys have no menstruation. It is by no means unlikely that the periodical secretion of the human uterus, which is, on the one hand, probably connected with the moral and physical constitution of the human female, on the other confers the capability of forming a placenta. The human female, after puberty, is always susceptible of sexual desire; to the permanency of which, unlike the periodic appetites of animals, it is probable that the menstrual secretion contributes. A uterus, with such large secreting powers as the human uterus, may be capable of pouring out decidua, and organizing part of it into a placenta; while the uteri of animals, habituated to no secretion, may on that account be capable of nothing more than increased capillary vascularity.

## Figures and Tables

**Figure f1:**